# Supplementary material for: An inverse Faraday effect generated by linearly polarized light through a plasmonic nano-antenna
Source: Nanophotonics. 2023 Jan 27;12(4):687–94. doi: 10.1515/nanoph-2022-0488 (PMC11636277; doi:10.1515/nanoph-2022-0488)
Supplement: Supplementary file 1 — Supplementary Material Details [file j_nanoph-2022-0488_suppl.docx]

Supplementary Material

**An Inverse Faraday effect generated by linearly polarized light through a plasmonic nano-antenna**

Xingyu Yang, Ye Mou, Romeo Zapata, Benoît Reynier, Bruno Gallas, and Mathieu Mivelle*

Sorbonne Université, CNRS, Institut des NanoSciences de Paris, INSP, F-75005 Paris, France

*Corresponding author: [mathieu.mivelle@sorbonne-universite.fr](mailto:mathieu.mivelle@sorbonne-universite.fr)

A list of the main content:

Supplemental Figures S1 and S2


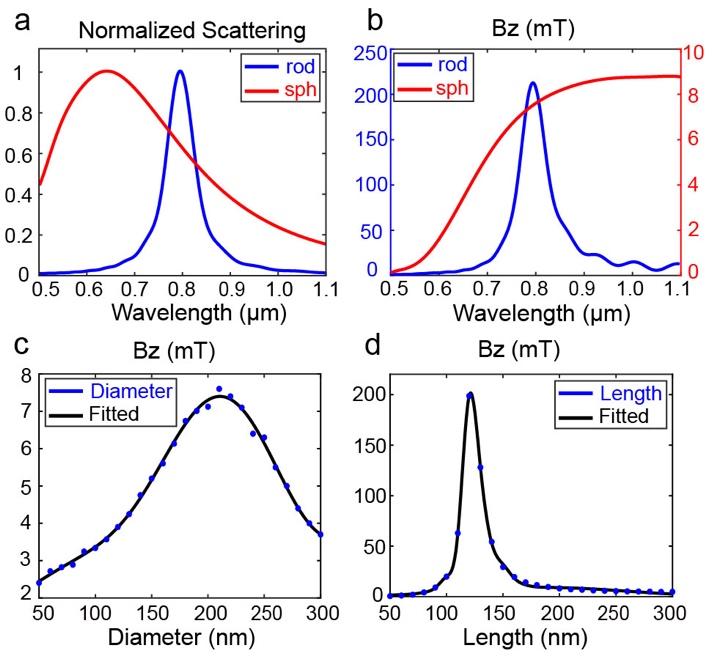


**Figure S1:** Optical and magnetic responses of gold nanoparticles and nanorods. Spectral responses of (A) the normalized scattering and (B) the magnetic field generated by the inverse Faraday effect for a gold nanoparticle of 210 nm in diameter (red curves) and a nanorod of 120 nm in length (blue curves). The magnetic field generated by inverse faraday effect for an excitation wavelength of 800 nm and a power density of 10^12^W/cm^2^ for (C) different diameters of gold nanoparticle and (D) different lengths of the nanorod.


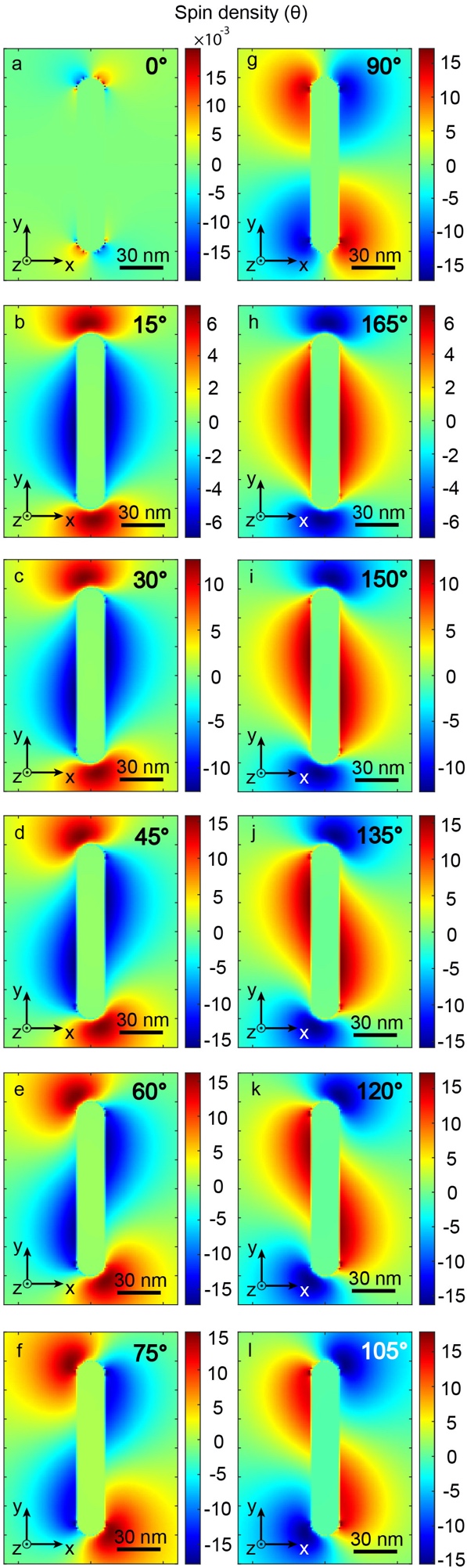


**Figure S2:** Near-field spin densities at the z-center of the gold nanorod in an xy transverse plane for different angles (in inset) of the incident linear polarization.
